# Moderately Hypofractionated Radiotherapy with Simultaneous Integrated Boost in Prostate Cancer: A Comparative Study with Conventionally Fractionated Radiation

**DOI:** 10.1155/2020/3170396

**Published:** 2020-11-28

**Authors:** Domenico Cante, Cristina Piva, Edoardo T. F. Petrucci, Piera Sciacero, Silvia Ferrario, Massimo Pasquino, Valeria Casanova Borca, Maria R. La Porta, Pierfrancesco Franco

**Affiliations:** ^1^Department of Radiation Oncology, ASL TO4, Ivrea Community Hospital, Ivrea, Italy; ^2^Department of Medical Physics, ASL TO4, Ivrea Community Hospital, Ivrea, Italy; ^3^Department of Oncology, Radiation Oncology, School of Medicine, University of Turin, Turin, Italy

## Abstract

**Background:**

To report 5-year clinical outcomes and toxicity in organ-confined prostate cancer (PCa) for low- and intermediate-risk patients treated with a moderately hypofractionated schedule of radiotherapy (RT) delivered with simultaneous integrated boost (SIB) compared to a conventionally fractionated RT regimen.

**Methods:**

Data of 384 patients with PCa treated between August 2006 and June 2017 were retrospectively reviewed. The treatment schedule consisted of hypofractionated RT (HYPO FR) with SIB up to 70 Gy to the prostate gland and 63 Gy to seminal vesicles delivered in 28 fractions or in conventionally fractionated RT (CONV FR) up to a total dose of 80 Gy in 40 fractions. Patient allocation to treatment was based on the time period considered. For intermediate-risk patients, androgen deprivation was given for a median duration of 6 months. The 5-year biochemical relapse-free survival (bRFS), cancer-specific survival (CSS), and overall survival (OS) were assessed. Furthermore, we evaluated gastrointestinal (GI) and genitourinary (GU) toxicities. Uni- and multivariate Cox regression analyses were used to test the impact of clinical variables on both outcome and toxicity.

**Results:**

A total of 198 patients was treated with hypofractionated RT and 186 with the conventional schedule. At a median follow-up of 5 years, no significant differences were observed in terms of GI toxicity and outcome between the two groups. Early GU toxicity was significantly increased in HYPO FR, while late GU toxicity was significantly higher in CONV FR. In HYPO FR, a biochemical relapse occurred in 12 patients (6.1%), and 9 patients (4.5%) reported a clinical relapse (4 local, 2 locoregional, and 3 systemic recurrence). In CONV FR, 15 patients (8.1%) experienced a biochemical relapse and 11 patients (5.9%) showed a clinical relapse (5 local, 4 locoregional, and 3 systemic recurrences). Early grades 1-2 GU and GI toxicities were observed in 60 (30.3%) and 37 (18.7%) patients, respectively, in the hypofractionated group and in 33 (17.7%) and 27 (14.5%) patients, respectively, in the conventionally fractionated RT group. Late GU and GI toxicities occurred in 1 (0.51%) and 8 (4.1%) patients, respectively, in HYPO FR. In CONV FR, 5 (2.7%) and 6 (3.2%) patients experienced late GU and GI toxicities, respectively. The 5-year OS, bRFS, and CSS were 98.9%, 94.1%, and 99.5%, respectively, in HYPO FR, and 94.5%, 92.1%, and 99.0%, respectively, in CONV FR.

**Conclusions:**

Results obtained in this study showed that moderately hypofractionated RT employing SIB can be an effective approach providing valuable clinical outcomes with an acceptable toxicity profile.

## 1. Introduction

In localized prostate cancer (PCa), the most common radiotherapy regimen consisted of 1.8- to 2-Gy fractions, delivered 5 days per week over 7–9 weeks. In the last decades, improvements in radiotherapy (RT) technologies combined with the radiobiological background of PCa led to a growing interest in moderate hypofractionation (2.5–4 Gy per fraction), and this approach has become an established treatment strategy [1–3].

Based on the linear quadratic model of radiation dose-response, it has been suggested that PCa has a lower *α*/*β* ratio (1.5 Gy) than bladder (6 Gy) and rectum (3 Gy) [4]. Thus, hypofractionation, employing a higher dose per fraction than those of conventional regimens, may result in a therapeutic advantage. Three large randomized trials demonstrated that moderate hypofractionated regimens (2.5 and 3 Gy per fraction) are not inferior to conventional schedules [5–7]. Beyond biology, the use of hypofractionation in PCa also has the advantage of improved patient convenience and higher cost-effectiveness [8].

Image-guided (IGRT) and intensity-modulated radiation therapy (IMRT) allow for dose escalation to a more conformal target volume limiting doses to organs at risk, resulting in a potentially lower toxicity [3].

In this study, we report on our monoinstitutional experience providing oncological outcomes and toxicity profile of hypofractionated and conventionally fractionated RT for the treatment of organ-confined PCa.

## 2. Materials and methods

### 2.1. Study Design, Radiotherapy Doses, and Volumes

Clinical data of prostate cancer patients who underwent image-guided static IMRT or volumetric arc therapy (VMAT) at our Institution between June 2006 and August 2017 were retrieved and collected. Two fractionation schedules were used: since 2009, a hypofractionated RT schedule (HYPO FR) consisting of 70 Gy delivered in 2.5 Gy/fraction along 28 fractions was adopted; prior to 2009, patients were treated with a conventionally fractionated schedule (CONV FR) consisting of 80 Gy given in 2 Gy/fraction over 40 fractions.

In the HYPO FR group, patients received 70 Gy to the prostate (Planning Target Volume 1-PTV1) and 63 Gy to the prostatic vesicles (PTV2) in 28 fractions with 2.5 and 2.25 Gy/fraction, respectively, delivered with a simultaneous integrated boost (SIB). In the CONV FR group, treatment consisted in 68 Gy delivered in 2 Gy/fraction to the prostate and vesicles (PTV2) for 34 fractions and a sequential boost given only to the prostate gland (PTV1) with 12 Gy delivered in 2 Gy/fraction. The intermediate-risk patients underwent androgen deprivation for a median duration of 6 months.

### 2.2. Planning and Imaging

All patients were treated in supine position with both legs immobilized. A preparation with an empty rectum and a full bladder was required. Five millimeter slice-thickness axial images of the pelvis were acquired. The Clinical Target Volume 1 (CTV1) corresponded to the prostate gland. The PTV1 was generated by adding a 5 mm margin around CTV in craniocaudal and posterior direction; an 8 mm margin was also added in the anterior and lateral directions. The CTV2 also included seminal vesicles, and the corresponding PTV2 was generated adding equivalent margins as aforementioned.

Bladder, rectum, small bowel, and femoral heads were separately contoured as organs at risk (OARs).

Plans were optimized using different Treatment Planning Systems (TPS) : Oncentra Masterplan v 3.0 (Nucletron, Venendhal, the Netherlands), Pinnacle3 (Philips), and Varian Eclipse (Varian®, Palo Alto, USA). For all patients, the dose was prescribed as the mean dose to the PTV1.

All patients received IMRT delivered using 6 MV photons. Daily Cone Beam Computer Tomography (CBCT) for the first five days of treatment and then once a week was performed, to ensure correct patient's positioning.

### 2.3. Follow-Up Protocol

Patients were followed up every 3 months for the first 2 years and every six months thereafter up to a 5 year observation time. Genitourinary (GU) and gastrointestinal (GI) toxicities occurring at any time-point were recorded. Those recorded during the course of the treatment or in the first 3 months were classified as early, otherwise as late. They were evaluated using the Common Toxicity Criteria for Adverse Effects (CTCAE) version 3.0 criteria. Biochemical recurrence, defined as 3 consecutive PSA rises, was reported. Clinical failures, confirmed by biopsy or imaging were classified into local (prostate or seminal vesicles), locoregional (pelvic lymph nodes), and systemic (distant metastasis). PSA values were recorded at the beginning of the therapy and at every follow-up evaluation.

### 2.4. Statistical Analysis

Mean and frequencies of the most relevant clinical variables were evaluated and reported for both groups. Differences between groups were evaluated using the *T* test, for continuous and normal variables, MannWhintney *U* test, for categorical variables, and chi-squared test for dicotomical variables. Overall survival (OS), cancer-specific survival (CSS), and biochemical relapse-free survival (bRFS) were calculated using KaplanMeier survival statistic, from the beginning of RT until death, for overall survival, until biochemical relapse for bRFS and until cancer recurrence for CSS. The impact of clinical variables, including age, androgen deprivation, Gleason Score (GS), TNM classification, and early gastrointestinal or genitourinary toxicities was evaluated using Cox proportional hazard model. A *p* value of 0.05 was considered statistically significant between groups. All statistical analyses were performed using Statistica 10 (StatSoft) software.

## 3. Results

### 3.1. Population Description

Clinical data of 384 patients were analyzed; 198 were comprised within the hypofractionated group and 186 within the conventionally fractionated group. Patients' characteristics are reported in Table 1. Following the indications of National Comprehensive Cancer Network v.3.2016 Classification, 76 and 83 patients were, respectively, identified as low-risk class (hypo and conventional), and 122 and 103 as intermediate-risk.

All 384 patients completed the treatment as planned and were alive at least 3 months after the end of radiation. Up to 66.2% of patients underwent static IMRT, whereas 33.9% received VMAT.  Table 2 shows differences between mean doses to organ at risk and PTV volumes between the two groups. PTV volumes and OAR max dose results show that groups were comparable.

### 3.2. Toxicity and Outcome

**Figure 1:**
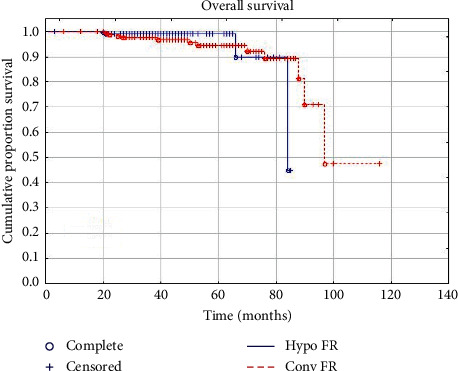
Overall survival.

**Figure 2:**
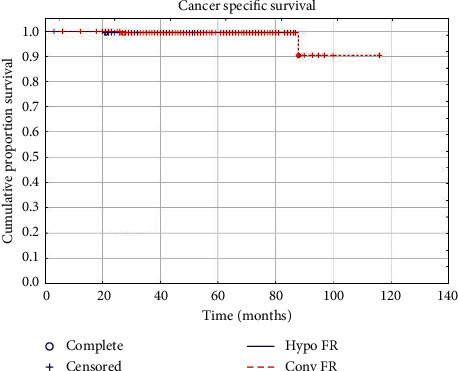
Cancer-specific survival.

**Figure 3:**
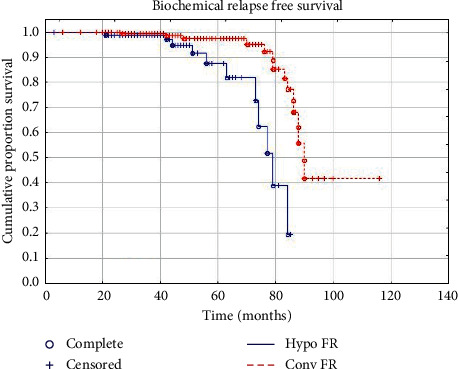
Biochemical relapse-free survival.

At a median follow-up of 55 months (range: 3–116 months), no significant differences were observed for GI toxicity and outcomes of biochemical control between the two groups. Early GU toxicity resulted significantly increased in HYPO FR, while late GU toxicity was significantly higher in the CONV FR group. A total of 13 patients died during observation-time due to other causes: specifically 3 patients in the HYPO FR group and 10 in the CONV FR. In HYPO FR, a biochemical relapse occurred in 12 patients (6.1%), whereas 9 patients (4.6%) reported also a clinical relapse (4 local, 2 locoregional, and 3 systemic recurrences). In CONV FR, 15 patients (8.1%) experienced a biochemical relapse and 11 patients (5.9%) showed also a clinical relapse (5 local, 4 locoregional, and 3 systemic recurrences). Early grade 1-2 GU and GI toxicities were observed in 60 (30.3%) and 37 (18.7%) patients, respectively, in the hypofractionated group and in 33 (17.74%) and 27 (14.52%) patients, respectively, in the conventionally fractionated RT group. Late GU and GI toxicities occurred in 1 (0.51%) and 8 (4.1%) patients, respectively, in HYPO FR. In CONV FR, 5 (2.7%) and 6 (3.2%) patients experienced late GU and GI toxicities, respectively. Detailed results are reported in Tables 3 and 4. The 5-year bRFS, CSS, and OS were 94.1%, 99.5%, and 98.9%, respectively, in HYPO FR, and 92.1%, 99.0%, and 94.5%, respectively, in CONV FR. Survival plots are reported in Figures 1–3. No clinical factors were found to be significantly correlated to biochemical control and survival.

## 4. Discussion

The current study, which compares moderate hypofractionation to conventionally fractionated RT for the treatment of organ-confined prostate cancer, reports clinical outcomes and toxicity with a mean follow-up of 5 years. Several randomized trials have been published with respect to the use of moderate hypofractionation versus conventional treatment, using various fraction sizes [5–7, 9–19]. Our fractionation schedule was chosen to deliver an equivalent 2 Gy dose expected to be at the same time effective in terms of tumor control and safe for OARs, similarly to the Cleveland Clinic experience [20]. Our results support the use of a moderate hypofractionated treatment using IGRT and IMRT with robust clinical outcome and an acceptable toxicity profile.

With respect to early toxicity, our overall rate of grade 1-2 GI toxicity was comparable between the hypofractionated group and the conventionally fractionated treatment (18.7% versus 14.5%). The overall rate of grade 1-2 GU toxicity was found to be higher in HYPO FR (30.3%) compared to CONV FR (17.8%) with statistical significance.

Our late toxicity data compare favourably with those of the available series on hypofractionated RT [9–12, 14, 17]. Our overall rates of ≥Grade 2 late GU toxicity were 0.51% and 2.7% in HYPO FR and CONV FR, respectively (*p* value = 0.002). Late GI toxicity occurred in 4.1% of patients belonging to the hypofractionated regimen (all grade 1 events) and in 3.2% of patients treated with the conventional schedule (6 grade-1 events and 1 grade-2), with no statistical significance. In the Cleveland Clinic experience, 770 men were treated with IMRT delivering 70 Gy in 2.5 Gy/fraction. The authors reported a 5-year rate of late grades 2-3 GI toxicity of 6% and 2%, respectively [20].

In the phase I-II study of Di Muzio et al. [21], intermediate- and high-risk patients were treated with 51.8 Gy to pelvic lymph nodes together with a concomitant simultaneous integrated boost to the prostate gland up to 74.2 Gy/28 fractions, whereas low-risk patients received 71.4 Gy/28 fractions to the prostate only. Overall rates of grade ≥3 late GU and GI toxicity were 5.9% and 6.3%, respectively.

In the 60 Gy hypofractionated cohort of a recent phase II trial [22], cumulative late grade ≥ 2 GI and GU toxicity at 8 years were 4% and 12%, respectively.

In 2017 Arcangeli et al. [23] published the final results of a phase III randomized trial that enrolled 168 patients with high-risk PCa who were randomly assigned to conventional (80 Gy in 40 fractions in 8 weeks) or hypofractionated RT (62 Gy in 20 fractions in 5 weeks) to prostate and seminal vesicles. After a median follow-up of 9 years, the rate of freedom from late ≥ grade 2 toxicity was 86% and 79% in the experimental and control arms, respectively, for GU toxicity and 86.5% and 84.6%, respectively, for GI toxicity, with no significant differences between the two treatment arms.

As literature data regarding clinical predictors of toxicity are few, we performed uni- and multivariate analysis with respect to all the considered prognostic variables; only early genitourinary and gastrointestinal toxicities showed a moderate correlation with the respective late toxicities endpoints in both hypofractionated and conventional fractionated groups.

With respect to tumor control and survival results, the most important findings of our study are the excellent outcomes in the two groups of treatment. No significant differences were observed in terms of biochemical and clinical relapses between the hypofractionated and conventional fractionated groups. Five years CSS and bRFS were 99.5% and 94.1%, respectively, for HYPO FR and 99% and 92.1%, respectively, for CONV FR. These results compare favourably with most studies on moderate hypofractionated RT [9–12, 14, 17–19]. Moreover, we reported a 5-year OS rate that was 98.9% and 94.5% in HYPO FR and CONV FR, respectively. Deaths observed during follow-up time could be attributed to prostate cancer in 3 patients (1 in HYPO FR and 2 in CONV FR) and to other causes in 13 patients (3 in HYPO FR and 10 in CONV FR).

In the Cleveland Clinic trial [20], the authors reported a 5-year bRFS of 94%, 83%, and 72% for low-, intermediate-, and high-risk patients, respectively.

In the study of Di Muzio et al. [21], the 5-year bRFS and CSS were 93.7% and 97.5%, respectively. They reported an OS of 88.6%.

In the recent phase II trial of Lieng et al. [22], at a median follow-up of more than 9 years, the 5- and 8-year freedom from biochemical failure (FFBF) for 60 Gy group was 81% and 66%, and for 66 Gy group, it was 88% and 80%.

In the phase III trial of Arcangeli et al. [23], the 10-year FFBF and CSS rates were 72% and 95%, respectively, in the hypofractionation group and 65% and 88%, respectively, in the conventional fractionation group. The authors showed 10-year OS rates of 75% and 64% in the hypofractionated and conventional fractionated groups, respectively.

In the present study, uni- and multivariate analysis did not result in any statistical appreciable correlation between all the considered prognostic variables and outcome results.

Probably, our favourable results in terms of outcome and toxicity can be attributed to several factors. First, the use of IMRT technique in all patients that allows highly conformal treatment plans to be delivered where the dose gradient is quite steep reducing dose to nearby OARs. Second, the use of IGRT provided us with the chance to reduce treatment margins and consequently toxicity profile associated to RT. Third, we have to consider that in these groups of patients the treatment of pelvic lymph nodes was not planned, with a potential benefit in terms of decreased toxicity.

The present study is a comparative single institution analysis that considered only one hypofractionated schedule; this can represent a limitation. Furthermore, toxicity results were evaluated using only a physician-rated toxicity scale (CTCAE version 3.0) with no patient-reported outcomes present or quality of life assessment. This would explain the obtained low toxicity incidence.

## 5. Conclusions

Our study confirms that moderately hypofractionated simultaneous integrated boost RT delivered using an image-guided protocol and static IMRT or VMAT technique is efficient and safe as it leads to a promising outcome with an acceptable toxicity profile. Our results complement other papers with a similar approach; a longer follow-up and the addition of other prospective data are needed to validate our results.

## Figures and Tables

**Table 1:** Patients characteristics.

Characteristics (*n* = 384 patients)	Hypo FR (*n* = 198 patients; 51.6%)	Conv FR (*n* = 186 patients; 48.4%)
Age, mean (std. dev)	74.4 years (±7.2)	72.2 years (±6.1)
Initial PSA (ng/ml), mean (range)	9.05 (0–564)	14.6 (3–618)
Gleason score		
7 (4 + 3)	54 (27.3%)	33 (17.7%)
7 (3 + 4)	53 (26.8%)	43 (23.1%)
6 (3 + 3)	89 (44.9%)	100 (53.8%)
≤5	2 (1%)	10 (5.4%)
Risk category		
Low risk	76 (38.4%)	83 (44.6%)
Intermediate risk	122 (61.6%)	103 (55.4%)
TNM		
T1	81 (40.9%)	80 (43%)
T2	117 (59.1%)	106 (57%)
Androgen deprivation		
Yes	154 (77.8%)	128 (68.8%)
No	44 (22.2%)	58 (31.2%)
Radiotherapy technique		
IMRT	103 (52%)	151 (81.2%)
VMAT	95 (48%)	35 (18.8%)

**Table 2:** Organs and targets data.

	Hypo FR (in EQD2^*∗*^)	Conv FR	*p* value
Rectum			
Mean dose (Gy) + std. dev	38.95 ± 9	40.04 ± 3	0.053
Max dose (Gy) + std. dev	69.20 ± 12	68.47 ± 14	0.010
Bladder			
Mean dose (Gy) + std. dev	29.29 ± 7	33.04 ± 9	0.002
Max dose (Gy) + std. dev	71.07 ± 21	68.83 ± 7	0.643
PTV1			
Mean vol. (cm^3^) + std. dev	161.97 ± 26	175.51 ± 18	0.435
PTV2			
Mean vol. (cm^3^) + std. dev	222.29 ± 34	223.62 ± 22	0.569

^*∗*^Equivalent doses delivered in 2-Gy fractions, calculated using *α*/*β* = 3 for rectum, *α*/*β* = 6 for bladder, and *α*/*β* = 1.5 for prostate cancer.

**Table 3:** Outcomes.

Outcome	Hypo FR-198 patients		Conv FR-186 patients		*p* value
Biochemical relapse	12	6.06%	15	8.06%	0.312
Clinical relapse	9	4.55%	11	5.91%	0.546
Local	4		5		
Locoregional	2		4		
Systemic spread	3		3		
Death					
Relapse	1	0.51%	2	1.07%	0.526
Other causes	3	1.52%	10	5.38%	0.036

**Table 4:** Toxicities.

Outcome	Hypo FR-198 patients		Conv FR-186 patients		*p* value
Gastronitestinal toxicities					

Early	37	18.69%	27	14.52%	0.273
G1	26		26		
G2	11		1		
G3	0		0		
G4	0		0		

Late	8	4.04%	6	3.23%	0.670
G1	8		4		
G2	0		1		
G3	0		1		
G4	0		0		

Genitourinary toxicities					

Early	60	30.30%	33	17.74%	0.003
G1	46		25		
G2	14		7		
G3	0		1		
G4	0		0		

Late	1	0.51%	5	2.69%	0.002
G1	0		5		
G2	0		0		
G3	1		0		
G4	0		0		

## Data Availability

The data supporting the results of the present study can be made available from the corresponding author upon request.
